# Accumulation Kinetics and Biological Action of Doxorubicin in Rabbit Intervertebral Discs

**DOI:** 10.3390/ijms26157386

**Published:** 2025-07-30

**Authors:** Eleni Mavrogonatou, Anastasios Kouroumalis, Lubna Khaldi, Christophoros Christophoridis, Dimitris Kletsas

**Affiliations:** 1 Laboratory of Cell Proliferation and Ageing, Institute of Biosciences and Applications, National Centre for Scientific Research “Demokritos”, 153 41 Athens, Greece; anastmalis@yahoo.gr (A.K.); c.christoforidis@bio.demokritos.gr (C.C.); 2 Department of Pathology, “Saint Savvas” General Anticancer Oncology Hospital, 115 22 Athens, Greece; lubna.khaldi@gmail.com; 3 Doping Control Laboratory of Athens, Institute of Biosciences and Applications, National Centre for Scientific Research “Demokritos”, 153 41 Athens, Greece

**Keywords:** annulus fibrosus, nucleus pulposus, cell viability, oxidative stress, senescence, in vitro, in vivo, collagen, proteoglycans

## Abstract

Doxorubicin (DOX) is widely used for the treatment of several tumors, but considerable dose-dependent side effects on many normal tissues, including bones, have been reported. The aim of the present study was to follow for the first time the kinetics of DOX accumulation/clearance in the non-vascularized intervertebral disc (IVD), as well as to assess the drug’s biological action in the annulus fibrosus (AF) and nucleus pulposus (NP) IVD cells and tissues. DOX was administered intravenously to rabbits before the isolation of IVDs, in which DOX quantification was performed using a highly sensitive LC-HRMS/MS analytical method. The effect of the drug on IVD cells’ physiology was assessed in vitro, while IVD tissue quality post-DOX administration was studied in vivo through histological analysis. DOX delivery was found significantly lower in the IVD compared to the highly vascularized skin, declining from the outer AF to the inner NP. The low DOX concentrations reaching the IVDs had marginal effects on cells’ viability, intracellular redox status, and p38 MAPK activation, while they did not evoke cellular senescence. Most importantly, the drug did not negatively affect ECM integrity, as collagen and proteoglycan content remained stable in vitro and in vivo.

## 1. Introduction

**Figure 1:**
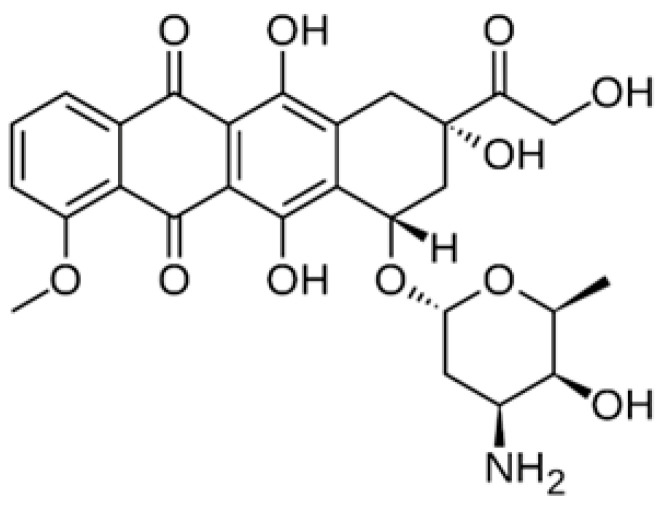
Chemical structure of doxorubicin (DOX).

Doxorubicin (DOX) (Figure 1) is a widely known DNA-damaging anti-tumor drug used in combination cocktails for the treatment of several malignancies, including breast, lung, thyroid and hematologic cancers [1]. However, considerable dose-dependent side effects have been reported by its use, such as cardiotoxicity [2] and systemic bone loss in children and adult cancer patients [3,4,5]. Even though numerous studies have addressed the effects of DOX on bone integrity, volume and thickness in humans and animal models [3,4,5,6,7,8], little is known yet on the effect of DOX specifically on vertebral bone quality [9,10,11] and even less on the effect of DOX on the intervertebral disc (IVD) [10], despite its integral position within the spinal column.

Intervertebral discs (IVDs) are the joints of the spine and consist of two main regions: the external annulus fibrosus (AF) and the centrally located nucleus pulposus (NP), surrounded on both sides by the cartilaginous endplates (CEPs) [12]. AF contains fibroblast-like cells and appears as a thick ring formed mainly by concentric type I collagen fibers, while NP is a highly hydrated gel containing chondrocyte-resembling cells and consisting mostly of type II collagen and proteoglycans [13,14]. IVD homeostasis is regulated by an exceptionally low number of cells, which are though responsible for maintaining the dynamic equilibrium between synthesis and degradation of the extracellular matrix (ECM). Low IVD cellularity has been attributed to the restrictive nutritional conditions prevailing in the tissue and deteriorating from the periphery to the center, since the absence of blood supply allows influx of nutrients and removal of metabolic by-products only via diffusion through the outer AF surface and the CEPs [13,15,16,17,18].

Apart from the nutritional confinement, local bioavailability of most systemically administered drugs or bioactive molecules within the IVD is also limited due to its avascular nature and the presence of endplate and synovial spaces [19,20,21]. Using a highly sensitive and reliable analytical method, we have recently shown that zoledronic acid—a bisphosphonate used in osteoporosis management—is rapidly and transiently accumulated in the blood plasma and the skin of a rabbit animal model, whereas it is slightly and belatedly accumulated or even not detected at all in the AF and the NP of the rabbit disc, respectively [13].

DOX accumulation has been studied in the past in various biological fluids and tissues, e.g., blood plasma and urine [22,23], kidney and liver [24], cancer tissues [25,26], brain, lung and heart [27]. Nevertheless, to the best of the authors’ knowledge, the DOX accumulation profile and kinetics in the IVD have not been studied thus far. Due to the distinct physicochemical environment of this tissue that allows limited access to the majority of molecules/compounds, the determination method requires additional pretreatment steps (tissue preparation and lysis) and the achievement of an extremely low limit of detection (LOD), in accordance with the anticipated low concentrations of the drug in the IVD of DOX-treated patients. Taking into account that DOX determination in complex biological matrices presents significant analytical challenges, an efficient sample treatment procedure would be necessary, preferably Solid Phase Extraction (SPE), with adequate chromatographic separation. Unequivocal identification and quantitative determination may be achieved using liquid chromatography-high resolution mass spectrometry (LC-HRMS)/MS, which provides increased precision and accuracy, even in complex sample matrices.

Given the reported negative effects of DOX on bones, the vicinity of IVDs to vertebrae and the lack of existing literature regarding the accumulation of the drug in IVDs, the aim of the current study was to assess the kinetics of DOX accumulation in the discs of a rabbit animal model. DOX detection and quantification were performed using a highly sensitive and accurate LC-HRMS/MS analytical method, while the kinetics of DOX accumulation in the IVD were compared to those of DOX accumulation in the plasma and the vascularized skin. Since DOX toxic effects have been reported to be exerted even in tissues displaying low drug delivery capacity (e.g., in the bones via DOX-induced oxidative stress [3]), determined DOX concentrations were then used in in vitro experiments to assess the effect of the drug on several parameters of rabbit IVD cells’ physiology, including cell viability, intracellular redox status, gene and protein expression, premature cellular senescence and ECM deposition. Finally, we investigated the effect of DOX administration on IVD tissue quality in vivo by histological analysis.

## 2. Results

### 2.1. Analytical Performance of Validated LC-HRMS Method

The analytical performance characteristics of the method developed for the determination of DOX in rabbit blood plasma, skin, AF and NP tissues are summarized in Table 1. The analytical figures of merit show that the accuracy and precision of the developed method are within acceptable regulatory limits. Figure S1 shows overlaid multiple reaction monitoring (MRM) chromatograms of the DOX calibration curve and the calculated calibration curve.

In the case of rabbit blood plasma, the linear dynamic range was found to extend from 5 to 200 nM DOX (R^2^ = 0.977), with high mean % recovery values (78.9%) and one-day repeatability ranging from 3.0 to 8.6%. The method was reproducible in two days, with a limit of detection (LOD) as low as 0.2 nM.

Determination of DOX in the rabbit skin demonstrated a linear dynamic range of 5–800 ng/g (R^2^ = 0.975) and a high mean recovery (79.9%). The LOD value was 3 ng/g. The method for DOX determination in the AF and NP was adequately accurate and precise, with a dynamic range of 5–200 ng/g (R^2^ = 0.944 and 0.982, respectively) and high repeatability and reproducibility characteristics.

### 2.2. Analysis of Rabbit Blood Plasma, Skin and IVD Samples

**Figure 2:**
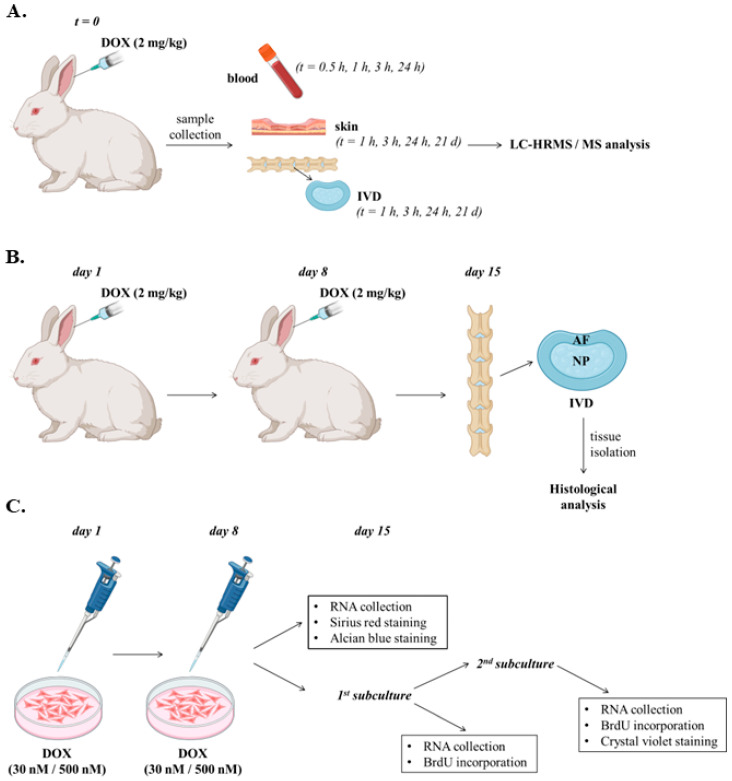
Graphical representation of doxorubicin administration protocols employed in in vivo and in vitro experiments of the current study. (**A**). Doxorubicin (DOX) was administered to rabbits intravenously through the ear vein at a dose of 2 mg/kg before blood plasma, skin and intervertebral disc (IVD) (annulus fibrosus, AF and nucleus pulposus, NP) isolation at the designated time-points, processing and preparation for LC-HRMS/MS analysis. (**B**). DOX was administered to the animals intravenously in two doses of 2 mg/kg on days 1 and 8. Untreated animals served as the control group. DOX-treated and control animals were euthanized at day 15 for tissue isolation and histological analysis. (**C**). AF and NP cell cultures were treated with 30 and 500 nM of DOX twice, with a 7-day interval, before gene expression analysis and assessment of collagen amount and proteoglycans’ content 7 days post-second DOX administration. For the assessment of DOX-induced cellular senescence, cells were subcultured once and twice (starting 7 days post-second DOX administration) before gene expression and cell proliferation analyses.

Treatment of animals with DOX, sample collection, as well as in vitro experiments were performed as summarized in Figure 2.

Initially, we tested the stability of DOX under the conditions of sample collection and processing, and it was found that the presence of collagenase, used for skin, AF and NP tissue lysis, did not affect its concentration (Figure S2).

**Figure 3:**
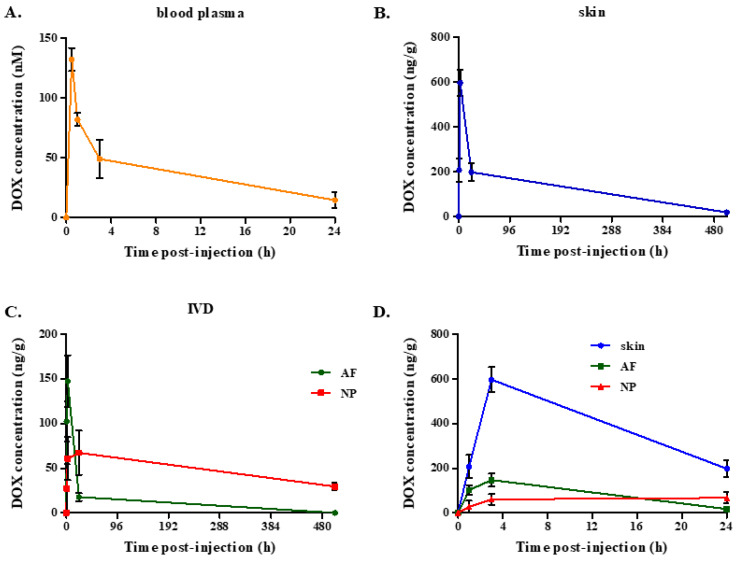
Kinetics of doxorubicin (DOX) accumulation in the blood plasma, skin and intervertebral disc (IVD) (annulus fibrosus, AF and nucleus pulposus, NP). DOX was administered to rabbits intravenously at a dose of 2 mg/kg before sample collection at the designated time-points, processing and liquid chromatography-high resolution mass spectrometry (LC-HRMS)/MS analysis. (**A**). Plasma was isolated by venous blood samples obtained from the ear that did not receive DOX. (**B**,**C**). Hairless skin and IVD tissue samples were isolated from DOX-treated and control animals. IVDs were separated from the bilateral vertebral bodies before NP and AF segregation. Tissue samples were collagenase-digested, centrifuged and supernatants were processed for LC-HRMS/MS analysis. (**D**). Merged representation of (**B**,**C**) at the time frame of 0–24 h post-injection, for the simultaneous monitoring/comparison of DOX accumulation in the vascularized skin and avascular IVD tissues. Averages ± standard deviations of three experiments are presented here.

For blood plasma samples, an initial abrupt increase in DOX concentration was observed (Figure 3A), reaching the maximum concentration of 132.4 ± 9.6 nM within 0.5 h post-administration. Within 3 h post-administration, only a DOX concentration of 49.1 ± 16.1 nM was determined.

Similar kinetics of DOX accumulation were observed in the skin, with the peak being recorded 3 h post-administration (Figure 3B). The maximal DOX concentration of 597.9 ± 57.0 ng/g decreased to 198.5 ± 39.0 ng/g within 24 h post-administration.

As mentioned above, DOX bioavailability in the IVD has not been assessed before. Interestingly, in the current study, differences in DOX accumulation were found in the IVD compared to the skin and plasma. In the AF, DOX concentration gradually increased during the first hours, reaching a maximum of 147.6 ± 29.0 ng/g 3 h post-administration (Figure 3C). Twenty-four hours post-administration, a residual low DOX accumulation in the AF (17.9 ± 4.5 ng/g) was still observed, but DOX was eventually entirely cleared from this tissue.

DOX was also detected in the inner part of the IVD, i.e., the NP, at a much lower concentration, which never returned to 0, even 21 days post-administration. In detail, DOX concentration in the NP was found to be approx. 60 ng/g 3 and 24 h post-administration, while a DOX concentration of 29.5 ± 4.0 ng/g was still detected 21 days post-administration (Figure 3C). Figure 3D summarizes the differences in the distribution of DOX accumulation in the vascularized skin and the avascular IVD tissues at the time frame of 0–24 h post-injection. DOX distribution was found to differ not only between the skin and the IVD, but also between the two distinct regions of the IVD, i.e., the AF and the NP.

**Figure 4:**
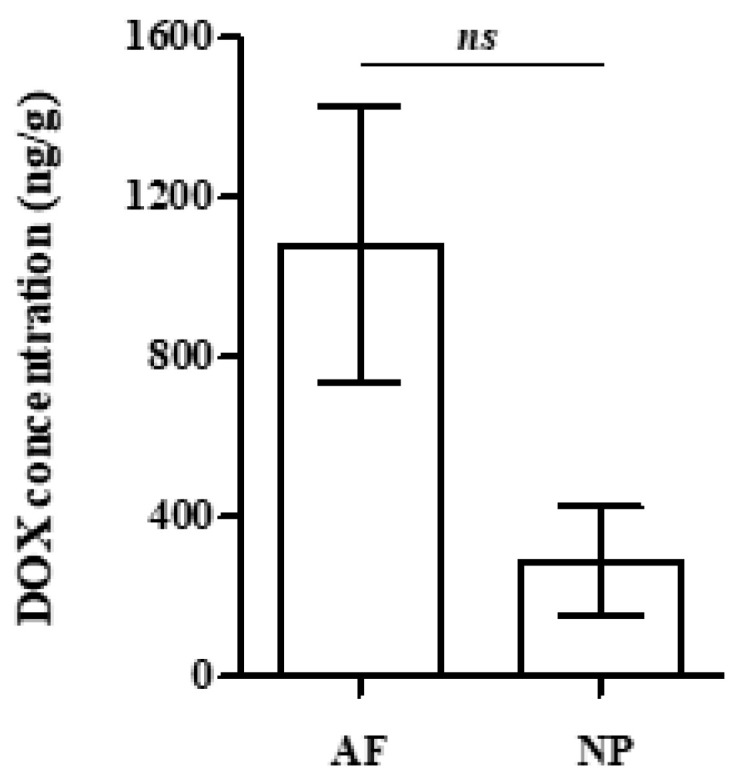
Uptake of doxorubicin (DOX) by annulus fibrosus (AF) and nucleus pulposus (NP) intervertebral disc (IVD) tissues immersed in a solution of DOX at the concentration of the rabbit plasma. In order to assess the uptake of DOX in tissues, AF and NP IVD samples post-immersion were treated with 2 mL of collagenase solution (3 mg/mL). The samples were left overnight under continuous shaking, were then centrifuged, and the supernatant was used for solid phase extraction (SPE) treatment and liquid chromatography-high resolution mass spectrometry (LC-HRMS)/MS analysis. No statistically significant differences were found between samples (Student’s *t*-test, ns: not significant).

To elucidate whether lower DOX accumulation in the NP was exclusively the result of its greater distance from the periphery of the IVD and the entire lack of vascularization or of its specific physicochemical composition compared to the AF, as well, isolated blank AF and NP tissues were separately immersed in DOX solution prior to the estimation of DOX uptake by each tissue. As shown in Figure 4, AF displayed a tendency for higher DOX accumulation in comparison to the NP, but this difference was not found to be statistically significant. This finding suggests that the accumulation of the drug rather depends on the tissues’ location and proximity to the blood supply than on the physicochemical environment of the different parts of the IVD.

Selected MRM chromatograms of the MS/MS transitions of DOX in the blood plasma, skin, AF and NP tissues are given in the Supplementary Material, Figures S3, S4, S5 and S6, respectively.

### 2.3. Effect of DOX on the Viability and Redox Status of Primary Rabbit AF and NP IVD Cells

**Figure 5:**
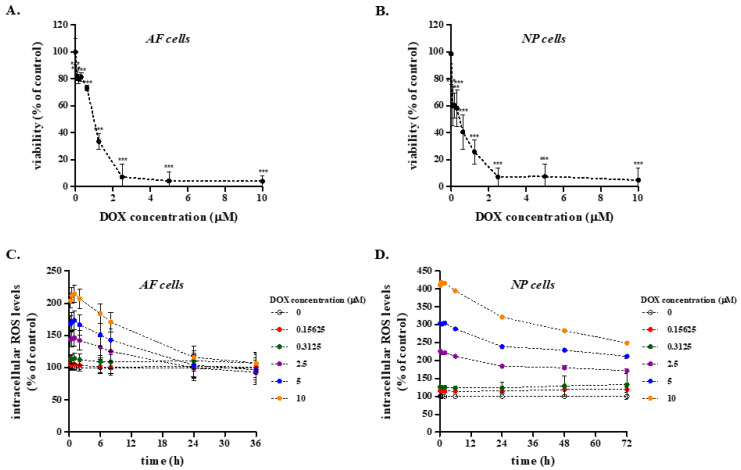
Effect of doxorubicin (DOX) on the viability and redox status of primary rabbit annulus fibrosus (AF) and nucleus pulposus (NP) intervertebral disc (IVD) cells. (**A**,**B**). Rabbit AF and NP IVD cells were plated in 96-well plates before the addition of DOX at concentrations from 0 to 10 μΜ for 72 h. Optical density of the solubilized formed MTT formazan crystals was measured at 550 nm and cell viability was calculated as a percent ratio of untreated cells. One representative experiment conducted in sextuplicate is depicted here, and the data presented are mean values ± standard deviations. Asterisks denote statistically significant differences compared to the untreated control (Student’s *t*-test, *** *p* < 0.001). (**C**,**D**). Rabbit AF and NP IVD cells were cultured in 96-well plates and incubated with 10 μM DCFH-DA for 1 h at 37 °C before their exposure to DOX (0–10 μΜ). Intracellular levels of reactive oxygen species (ROS) were estimated by recording fluorescence (excitation wavelength: 485 nm, emission wavelength: 520 nm). ROS production was expressed as a % ratio of the untreated control. Experiment was repeated three independent times, and one representative graph is shown here.

We then assessed the effect of a wide range of DOX concentrations (0–10 μM) on the viability of primary rabbit IVD cells in vitro using the MTT assay. As shown in Figure 5A,B, DOX resulted in a dose-dependent reduction in AF and NP cell viability, with median lethal doses (LD_50_) of 1.287 ± 0.437 μM and 0.533 ± 0.258 μM, respectively, that far exceed the highest concentration measured in the rabbit plasma and the IVD tissues.

Furthermore, DOX was not found to increase intracellular ROS levels in primary rabbit AF and NP cells at concentrations lower than 312.5 nM and a more pronounced DOX-induced oxidative stress in these cells was only observed at concentrations that were equal to or higher than the respective LD_50_s, which, as mentioned above, are much greater than DOX concentrations measured in the rabbit plasma and even greater than those of the distant from the vasculature IVD tissues (Figure 5C,D).

Next, we studied the effect of the residual in the plasma DOX concentration on the activation of major stress-activated intracellular signaling pathways. After a single treatment with the residual in the plasma DOX concentration, i.e., 30 nM, only a slight p38 MAP kinase (MAPK) activation was observed (Figure S7), implying that a low DOX concentration imposes a mild stress to the cells, which is most probably rapidly counteracted. The absence of severe stress induced by a low DOX concentration in IVD cells was confirmed by the absence of activation or overexpression of proteins known to be implicated in biochemical pathways that regulate cell viability, proliferation and stress responses (i.e., extracellular signal-regulated kinases, ERKs and c-Jun N-terminal kinases, JNKs), as well as of the cell cycle regulators p53 and p21^WAF1^. On the other hand, phosphorylation or overexpression of all tested proteins was observed—as anticipated—when cells were treated with the supraphysiological DOX concentration of 500 nM, which served as a positive control (Figure S7).

### 2.4. Effect of DOX on the Transcriptional Profile of Selected Genes in Rabbit AF and NP IVD Cells

**Figure 6:**
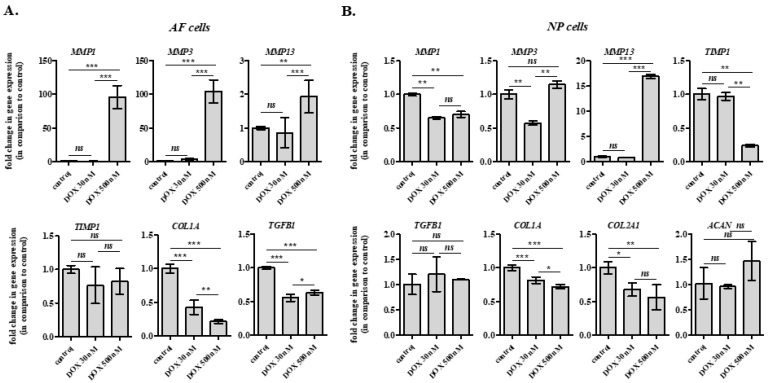
Doxorubicin (DOX) concentration prevailing in the plasma slightly affects the transcriptional profile of rabbit annulus fibrosus (AF) and nucleus pulposus (NP) intervertebral disc (IVD) cells in vitro. AF (**A**) and NP (**B**) cells were treated twice with 30 or 500 nM DOX with a 7-day interval before sample collection and RNA extraction 7 days post-second DOX administration. Extracted RNA was then used for RT-qPCR analysis, with glyceraldehyde-3-phosphate dehydrogenase (GAPDH) serving as the reference gene. Numerical values are the means ± standard deviations of at least two independent experiments conducted in duplicate. Asterisks denote statistically significant differences between any pair of means (ANOVA, Tukey’s test, * *p* < 0.05, ** *p* < 0.01, *** *p* < 0.001, ns: not significant).

To further elucidate the effect of DOX on IVD cells, we assessed putative alterations in mRNA levels of selected genes in rabbit AF and NP cells in vitro following the administration scheme of the drug that we used in vivo. In detail, cells were treated twice with 30 nM DOX with a 7-day interval before RNA extraction 7 days post-second DOX administration (Figure 2C). The same protocol was also followed using the much higher DOX concentration of 500 nM. Given that AF and NP IVD cells are the ECM producers of the IVD tissue, we focused on the transcriptional profile of genes encoding ECM components (i.e., type I collagen, type II collagen and aggrecan) and ECM-degrading molecules [i.e., matrix metalloproteinases (MMPs)] or their regulators [i.e., transforming growth factor β1 (TGF-β1) and tissue inhibitor of metalloproteinase 1 (TIMP1)]. In AF IVD cells, DOX at the concentration of 30 nM was not found to alter MMP1, MMP3 nor MMP13 mRNA levels, in contrast to the concentration of 500 nM that induced all MMPs’ up-regulation (Figure 6A). On the other hand, type I collagen and TGF-β1 mRNA levels were found to be down-regulated in DOX-treated AF IVD cells even at the concentration of 30 nM (Figure 6A). In NP IVD cells, a slight down-regulation of type I and II collagens was induced by both DOX concentrations (Figure 6B). However, no MMPs’ up-regulation and no changes in TIMP1 mRNA levels were observed at the concentration of 30 nM, in contrast to the pronounced up-regulation of MMP13 and down-regulation of TIMP1 induced by the concentration of 500 nM (Figure 6B).

### 2.5. Long-Term Effects of DOX Administration on IVD Cells

**Figure 7:**
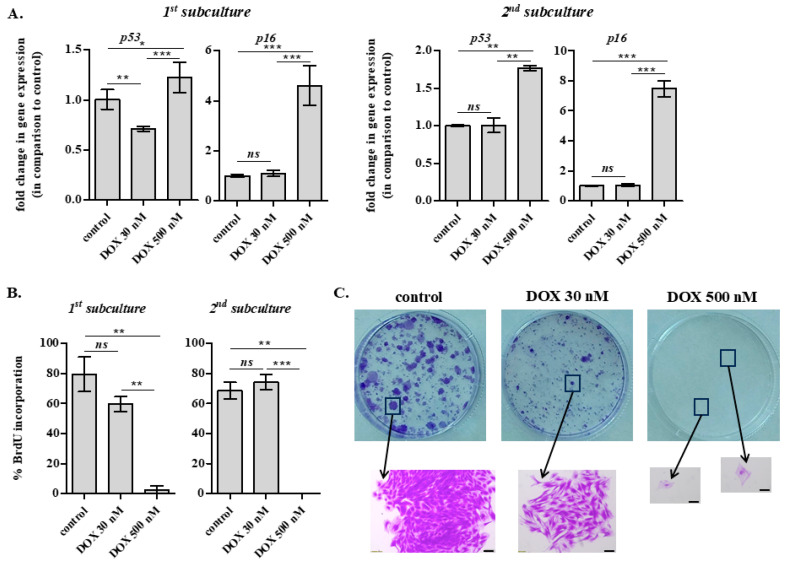
Doxorubicin (DOX) concentration prevailing in the plasma does not result in the induction of IVD cells’ senescence in vitro. DOX 30 and 500 nM were administered to rabbit annulus fibrosus (AF) intervertebral disc (IVD) cells once per week for two weeks, and 7 days post-second DOX addition, cells were subcultured once or twice. (**A**). RNA was extracted 7 days post-subculture and RT-qPCR analysis for p53 and p16^INK4a^ (p16) was performed, with glyceraldehyde-3-phosphate dehydrogenase (GAPDH) serving as the reference gene. (**B**). Cells exposed to DOX treatment following the same protocol were plated onto coverslips before their incubation with bromodeoxyuridine (BrdU), fixation and immunofluorescence analysis for the estimation of BrdU incorporation. Numerical values are the means ± standard deviations of at least two independent experiments conducted in duplicate. Asterisks denote statistically significant differences between any pair (ANOVA, Tukey’s test, * *p* < 0.05, ** *p* < 0.01, *** *p* < 0.001, ns: not significant). (**C**). Rabbit AF IVD cells after the second subculture were sparsely plated in 6-well plates. Cells were stained with Crystal violet two weeks later. Scale bar: 200 μm.

We then followed by investigating the possibility of long-term senescence induction in rabbit IVD cells in vitro. For that reason, AF IVD cells were exposed to 30 and 500 nM of DOX twice, once per 7 days, and were then subcultured twice, with the first subculture being performed 7 days post-second DOX administration (Figure 2C). Cells were further incubated for another 7 days after each subculture before RNA extraction or immunofluorescence analysis for the estimation of bromodeoxyuridine (BrdU) incorporation. As shown in Figure 7A, 30 nM of DOX was not found to cause a long-term cell cycle arrest and even more senescence, since no up-regulation of p53 and p16^INK4a^ gene expression was observed, in contrast to what happened when cells were exposed to 500 nM of DOX.

In accordance, while 500 nM of DOX resulted in a complete loss of AF IVD cells’ proliferative capacity even from the first subculture after DOX treatment (shown by their scant, almost null nuclear BrdU incorporation), 30 nM of DOX provoked a transient small reduction in proliferation, but cells regained their proliferative potential in the progress of time (Figure 7B). This was also confirmed by the colony formation assay performed in DOX-treated cells (Figure 7C). As anticipated, the assay revealed an entire abolishment of cell proliferation at the concentration of 500 nM, where solely scattered single, non-proliferating cells could be observed under the microscope, which—in combination with the aforementioned p16^INK4a^ up-regulation—would support the induction of senescence. On the other hand, a similar number of—smaller in size—colonies was observed at 30 nM of DOX in comparison to control cells, evidencing an initial delay, but not a definite cessation of cells’ proliferation.

### 2.6. Effect of DOX Administration on ECM Quality of the Rabbit AF and NP In Vitro and In Vivo

**Figure 8:**
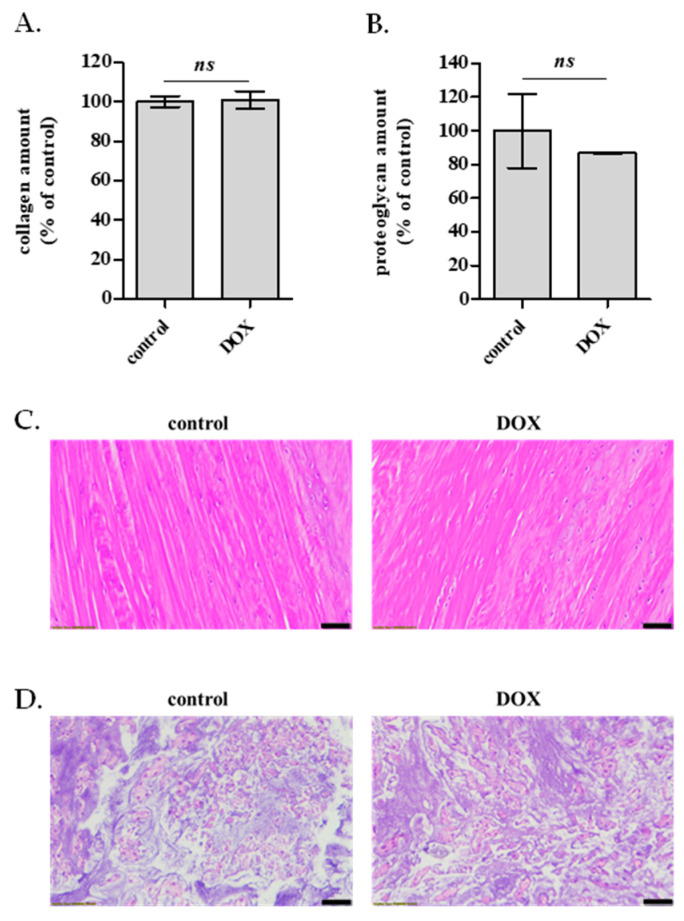
Effect of doxorubicin (DOX) on rabbit annulus fibrosus (AF) and nucleus pulposus (NP) extracellular matrix (ECM) quality in vitro and in vivo. (**A**). Collagen deposition in rabbit AF cells cultured in the absence (control) or the presence of DOX (30 nM) administered twice with a 7-day interval was assessed with Sirius Red staining 7 days post-second DOX administration. Quantification of solubilized collagen-bound Sirius Red stain was performed by measuring the absorbance at 540 nm. (**B**). Glycosaminoglycans’ content in rabbit NP IVD cells cultured in the absence (control) or the presence of DOX (30 nM) administered twice with a 7-day interval was assessed with Alcian Blue staining 7 days post-second DOX administration. Quantification of the extracted Alcian Blue stain was performed by measuring the absorbance at 620 nm. Collagen amount and glycosaminoglycans’ content normalized to cell number were expressed as a % ratio of the control. Mean values ± standard deviations of a representative experiment conducted in triplicate are demonstrated. No statistically significant differences were observed compared to the untreated control (Student’s *t*-test, ns: not significant). (**C**,**D**). Five μm histological sections of formalin-fixed and paraffin-embedded rabbit AF and NP IVD tissues, derived from the lumbar spine of animals injected twice with 2 mg/kg DOX 7 days post-second DOX injection, were mounted on microscopic slides and stained with hematoxylin or eosin. AF (**C**) and NP (**D**) IVD sections were examined under transmitted light microscopy. Representative digital photos obtained using ×20 objective lenses are shown here. Scale bar: 50 μm.

Given that the primary components of the AF and NP ECM are collagen and proteoglycans, respectively, their content in rabbit AF and NP IVD cells exposed to DOX treatment as described in Figure 2C was estimated in vitro. Sirius red staining was performed to estimate the effect of DOX on the amount of deposited collagen by primary AF cells, while Alcian Blue staining was performed to estimate glycosaminoglycans’ content of DOX-treated NP cells in vitro. Both the intensities of Sirius Red and Alcian Blue staining, normalized to cell number, were found to be unaltered in cells treated with 30 nM of DOX (Figure 8A,B). This observation was confirmed in vivo after the histological analysis of the AF and NP IVD tissues of untreated and DOX-treated rabbits, according to Figure 2B, which revealed no differences in tissue quality (Figure 8C,D).

## 3. Discussion

Anthracycline glycoside antibiotics are potent anticancer agents, widely used for the treatment of several types of malignancies, which classifies them within the list of essential medicines according to the World Health Organization [28]. However, despite the large number of clinical therapeutic protocols relying on the use of anthracyclines against adult and infant neoplastic diseases, adverse side effects on normal organs and tissues have been often recorded, a major one being cardiotoxicity [28,29]. Doxorubicin (DOX) or Adriamycin is a naturally occurring anthracycline routinely used as a chemotherapeutic agent against lymphomas, leukemias and solid tumors [30]. Given that its anticancer action is ascribed to universal cytotoxic mechanisms such as DNA intercalation and alkylation, interference with RNA and DNA polymerase, inhibition of topoisomerase II and ROS production [30,31], it is not surprising that DOX biodistribution has long been extensively studied.

DOX concentration in the plasma, as well as DOX accumulation in organs and tissues has been estimated in the past in humans and several animal models, using various drug administration schemes and diverse detection methods: analyses have been performed in clinical samples of patients [32,33,34] and experiments have been conducted in mice [30,35], rats [36,37,38], rabbits and guinea pigs [39]; single or repetitive DOX doses have been assessed, administered by intravenous or intraperitoneal injections [35,37,38,40]. DOX levels have been traditionally quantified by HPLC with fluorimetric detection [35], by HPLC validated with histologic image analysis of the harvested tissues [41] and more recently by LC-MS/MS analysis [38]. DOX distribution, kinetics of elimination and toxic effects have been experimentally determined or mathematically predicted in the majority of tissues and organs of the body, including the heart, the liver, the kidney, the lung, the spleen, the intestine, the omentum, the abdominal wall, the skin, the muscles, the bone marrow, the bile, the diaphragm and the bladder [30,35,36,38,40,41,42,43].

Amongst DOX side effects, impairments in bone development and quality have been reported for humans [3], as well as for rats and rabbits [6,8,44]. Furthermore, DOX exposure has been shown to result in rabbit articular chondrocytes’ apoptosis and to inhibit chondrocyte differentiation in vitro, as well as to exacerbate osteoarthritis (OA) progression and to provoke chondrocyte inflammation and apoptosis in a destabilization of the medial meniscus (DMM)-induced OA mouse model [45,46,47]. Taking into account that evidence on the bioavailability and effects of DOX on IVD homeostasis has been scarce thus far, our aim in the present study was to follow the kinetics of DOX accumulation/elimination—administered in doses that were in accordance with previous clinical studies in different types of cancer [48,49]—in the AF and NP IVD tissues. Therefore, we employed a state-of-the-art LC-HRMS method using a rabbit model, as rabbits have long served as an appropriate experimental model for the in vivo study of IVD degeneration [50,51,52,53]. Animals were treated by a clinically relevant regimen of weekly low DOX concentration doses, intravenously administered [54,55,56], which was preferred over intraperitoneal injections to avoid inflammation and subsequent chemical peritonitis [35].

DOX quantification in rabbit plasma and three tissues (i.e., skin, AF and NP IVD tissues) following a single intravenous dose of 2 mg/kg was determined at different time-points by our fully validated, reliable and specific LC-HRMS/MS method. Time-points of tissue collection were selected in order to cover extended kinetics, from very early (i.e., 0.5 or 1 h) to very late (i.e., 21 days) time-points, using the minimum number of animals. The proposed method offered good repeatability and high sensitivity (limit of quantification, LOQ < 1 nM or 20 ng/g), allowing the monitoring of DOX pharmacokinetics using small-volume samples. DOX intravenous administration resulted in a quickly achieved high DOX plasma concentration, followed by an initial rapid decline and an ensuing slower decrease, with very low DOX levels being retained 24 h post-injection, in accordance with previous studies [32,38,57,58,59]. DOX’s fast decrease in the plasma has been attributed to a rapid and extensive distribution into several tissues, including the kidney, liver and lung, all rapidly accumulating and then slowly releasing DOX [32,35,38,42].

We then assessed DOX biodistribution in the AF and NP IVD tissues (which, to the best of our knowledge, has not been estimated before) in comparison to the skin, a tissue in which DOX bioavailability has been quantified in previous studies [41,42]. The same extraction protocol was used for both AF and NP tissues, with higher inter-day variability being observed in the NP matrix that could be ascribed to the heterogenous, dense, gelatinous nature of this particular tissue. In addition, given the much smaller surface area of the NP within each separate IVD, the tissue quantity required for the analysis (100 mg) was obtained from a higher number of IVDs, which increased the variability of the starting material. DOX accumulation in the AF followed a similar kinetics profile to that followed in the skin, i.e., DOX concentration quickly increased (within 1 h post-injection) and reached its peak value (which was approx. 4-fold lower than the respective value quantified in the skin) 3 h post-injection, before the onset of the drug’s elimination. In addition, total DOX clearance was observed in the AF, similar to the skin. The rapid clearance of DOX from the skin is most possibly attributed to its vascular nature, which enables the fast transport of DOX into and out of the tissue. Thus, differences in DOX bioavailability in the AF compared to the skin could be attributed to the fact that AF is largely avascular, with only its outer part being in proximity to the vascular network, allowing DOX transportation into and out of the tissue mainly via diffusion. On the other hand, even though similar initial DOX accumulation kinetics were found for AF and NP, DOX peak concentration in the NP (reached 3 h post-injection) was found to be approx. 10- and 2.5-fold lower than the respective values in the skin and the AF and the drug was not found to be entirely released from the tissue even 21 days post-injection. Our findings on DOX poor delivery in the proximal to the bones AF and NP tissues within the spine are in accordance with our previous study reporting low bioavailability of zoledronic acid in the IVDs, as well [13]. This information could be valuable in clinical practice, given that a combinatorial therapy of zoledronic acid and DOX has been proposed for the efficient treatment of breast tumor metastases in bones [33].

Different kinetics of DOX accumulation/elimination in AF and NP IVD tissues in comparison to the skin could be ascribed to multiple factors such as dissimilar access to the blood flow, diffusion coefficients, lipid solubility, ECM composition and binding affinity or even cell density, given the drug’s covalent and non-covalent DNA-binding ability [30]. Indeed, AF and NP IVD tissues display limited accessibility compared to the highly vascularized skin: only the outer part of AF is in proximity to the vascular network, while NP is entirely avascular and distant from blood supply [60,61], allowing DOX transportation into and out of the tissues solely via passive diffusion. Furthermore, sulphated proteoglycans’ ability to bind water-soluble DOX has been long ago reported [62], providing a possible explanation for the drug’s slow release and prolonged elimination period observed in the proteoglycan-rich NP tissue [13,14]. Finally, diminished DOX concentrations attained in the IVD tissues in comparison to the skin may also be attributed to the extremely low cellularity—and thus low amount of nuclei per gram of tissue—in the AF, further declining towards the NP [63], since DOX tissue distribution has been previously positively correlated with tissue DNA concentration in rabbits [39]. To further elucidate differences in DOX delivery between AF and NP, isolated AF and NP tissues were immersed in the same DOX solution and DOX adsorption was quantified. In this blood vessel-free environment, DOX uptake by the NP displayed only a tendency to be inferior compared to the AF, suggesting that variations in DOX accumulation between AF and NP are mainly ascribed to their distance from vasculature and, to a lesser extent, to the tissues’ different physicochemical properties. Although DOX clearance was not directly tested using this experimental setup, this finding would also explain the persistence of DOX in the more distant from the vasculature NP, resulting in the retention of residual DOX levels within the tissue 21 days post-treatment.

Subsequently, we explored the direct effect of DOX on rabbit primary AF and NP IVD cells’ viability, which—to the best of our knowledge—has not been investigated before. We showed that NP cells were more sensitive than AF cells to high DOX concentrations, but no effect was observed on AF nor NP cells’ viability at DOX concentrations close to the residual in the animal’s plasma DOX concentration several hours post-injection (approx. 30 nM). Given that DOX has been shown to be mainly cardiotoxic via the induction of oxidative stress [64] and to impair bone integrity in cancer patients via oxidative stress [3], as well as to suppress chondrocyte differentiation by stimulating ROS production [46], the effect of a range of DOX concentrations on the redox status of rabbit AF and NP IVD cells was also tested. DOX resulted in elevated intracellular ROS levels in both AF and NP IVD cells only at concentrations that far exceeded the one estimated in the rabbit plasma. In accordance with the absence of DOX-induced cytotoxicity and oxidative stress, no induction of biochemical pathways regulating cell viability/proliferation (p53 and p21^WAF1^) was observed, while from the three members of the stress-regulated MAPK superfamily (i.e., ERKs, JNKs and p38 MAPK), only p38 MAPK was slightly activated.

To assess the long-term effects of DOX at the cellular level in vitro, we employed the two-dose DOX administration scheme (using the residual DOX concentration measured in the rabbit plasma, i.e., 30 nM). The transcriptional profile of selected genes encoding molecules related to ECM homeostasis was studied in DOX-treated AF and NP IVD cells, while typical markers of cellular senescence were investigated using a broader time frame to explore the possibility of DOX stress-induced premature senescence (SIPS). Both AF and NP cells treated with 30 nM of DOX displayed some individual transcriptional changes (i.e., decreased type I collagen, type II collagen and TGF-β1 mRNA levels), but hardly approached the profoundly catabolic phenotype of cells treated with 500 nM of DOX. In addition, in contrast to cells exposed to 500 nM of DOX, which were found to entirely lose their proliferative potential and to up-regulate p53 and p16^INK4a^ (the latter being an established biomarker of cellular senescence, in line with other cell models [65]), 30 nM of DOX resulted in an initial transient cell cycle delay, but not an irreversible arrest. This observation is contradictory to cellular senescence reported for vascularized tissues, such as the kidney, the liver [66], and the heart [67], possibly explained by the much higher DOX delivery that has been shown in these tissues. Most importantly, no effect of DOX was observed on collagen amount and proteoglycan content of DOX-treated AF and NP cells, respectively, in vitro. These findings were confirmed in vivo by the histological analysis of the AF and NP of IVDs derived from the lumbar spine of DOX-treated rabbits that displayed no difference in tissue quality compared to the IVDs of untreated animals, in contrast to DOX-induced IVD defects previously reported in rats [10]. This discrepancy could be attributed to the different species (rat vs. rabbit), to the different mode and duration of drug administration (intraperitoneal vs. intravenous injection, sacrifice on day 38 vs. day 15) and most importantly to the different ages (neonate animals with developing IVDs vs. older animals with mature IVDs).

In conclusion, here we used a well-validated, reproducible and reliable LC-HRMS/MS methodology, by which we determined DOX accumulation/elimination kinetics in the AF and NP IVD tissues of a rabbit model for the first time. We showed that DOX delivery is lower in the IVD compared to the highly vascularized skin, with the drug’s uptake declining from the outer AF towards the inner NP, mainly due to the latter’s greater distance from the blood supply. Low DOX concentrations reaching the IVDs were shown to have marginal effects on cellular physiology, as well as ECM homeostasis in vitro, translating into the maintenance of an unaltered tissue quality in vivo, at least for the DOX administration scheme applied here and the tested time frame. Further investigation is needed by extending the number of DOX injections/doses to better simulate anti-cancer regimens, by prolonging the time span of observation and by including degenerated—and thus accessible to vessels—IVDs. Scaling our method to human tissue could possibly be challenged by species-specific differences in the biochemical composition and structure of the IVDs, affecting DOX biodistribution. On the other hand, scaling in detecting DOX metabolites would require new matrix-matched calibration curves and optimization of our method for each species, as dissimilarities in produced metabolites would be expected due to the potentially different, species-specific metabolic pathways. Our findings give prominence to the requirement for bioavailability analysis of any traditional or newly discovered drug in additional tissues/organs beyond its specific target, which may find application in clinical practice in order to confine undesirable collateral side effects. Finally, the method of chemical analysis employed here could prove useful in the future—undergoing modifications/adaptations dependent on the molecular properties of a given investigated drug—to assess and/or validate the intentional successful delivery of pharmacological agents in the IVD, e.g., of agents that need to reach the tissue in order to prevent IVD degeneration and low back pain.

## 4. Materials and Methods

### 4.1. Chemical Reagents

In vitro and in vivo experiments in the current study were conducted with doxorubicin hydrochloride purchased in its pharmaceutical form, as a solution for injection (Adriamycin^®^ 50 mg/25 mL, Pfizer Inc., Manhattan, NY, USA). For the development of the analytical method, doxorubicin hydrochloride (>98%) was purchased from Merck (Darmstadt, Germany). Solid phase extraction cartridges (HyperSep™ C18 Cartridges, 3 mL, 200 mg) were obtained from Thermo Fisher Scientific (Darmstadt, Germany). Methanol (MeOH) of HPLC grade (99.99%) was obtained from Fischer Scientific (Leicestershire, UK), acetonitrile (ACN) of LC-MS grade was obtained from Sigma-Aldrich (St. Louis, MO, USA). High purity water (18.2 M) was produced on-site using a Millipore water purification system (Billerica, MA, USA). Formic acid (FA) (LC-MS grade) was purchased by Riedel-de Haën (Seelze, Germany). Methylthiazolyldiphenyl-tetrazolium bromide (MTT) was supplied by Merck. 2′,7′-dichlorodihydrofluorescein diacetate (DCFH-DA), Crystal Violet, Sirious Red F 3B (Direct Red 80) and Alcian Blue 8GX were obtained from Sigma-Aldrich. Real-time PCR primers were purchased from Eurofins Genomics (Ebersberg, Germany). Oasis HLB (200 mg, 6 cc, 25–35 μm, Waters Corporation, Milford, MA, USA) was used for SPE.

### 4.2. Standards and Solutions

DOX stock solution in MeOH:water (50:50) 1000 mg/L was prepared and stored at 4 °C. Stock standard solutions at 1 and 10 mg/L were also prepared in ultrapure water. For the determination of DOX in water and rabbit plasma, working standard solutions of 1, 2, 5, 10, 50 μg/L were prepared in ultrapure water and blank rabbit plasma, respectively. For the determination of DOX in skin, AF and NP tissues, working concentrations of 1, 2, 5, 10, 50 ng/mg were prepared, with adequate spiking of stock to blank tissue samples. QCs were prepared at concentrations of 0.5, 5 and 20 μg/mL in blank plasma, and at concentrations of 1 and 10 ng/mg for other tissues and stored at −80 °C.

### 4.3. Animals and DOX Treatment

Twenty male, 3-month-old New Zealand white rabbits (*Oryctolagus cuniculus*) of a mean weight of 4 kg were used in this study. All animals were retained in steel cages and conventionally bred in the Animal House of the Institute of Biosciences and Applications, NCSR “Demokritos”, where they were daily checked regarding their health status. This study was authorized by the Veterinary Department of the Attica Prefecture (license no: 132698/01.02.2023) and the NCSR “Demokritos” Research Ethics Committee (license no: 18/06.03.2023). For the assessment of DOX distribution in the blood plasma, the skin and the IVD (AF and NP), DOX diluted in saline buffer was administered to the animals intravenously (slowly through the ear vein) at a dose of 2 mg/kg before sample collection at several time-points (Figure 2A). For the assessment of the long-term effects of DOX on IVD tissues, DOX diluted in saline buffer was administered to the animals intravenously (through the ear vein) in two doses of 2 mg/kg at days 1 and 8, each injection lasting for at least 15 min (Figure 2B). This dosage and administration scheme was in accordance with previous clinical studies using patients with different types of cancer [48,49] and similar to the published regimens in rabbit and other animal models [33,68,69,70,71]. Untreated animals served as the control group. DOX-treated and control animals were euthanized for tissue isolation at day 15.

### 4.4. Plasma Isolation

Venous blood samples (approx. 5 mL) were obtained from the ear that did not receive DOX at 0, 0.5, 1, 3, and 24 h post-DOX injection, as previously described [13]. Blood samples were centrifuged at 1500× *g* for 30 min at room temperature and the supernatant plasma was stored at −80 °C until analysis.

### 4.5. Rabbit Skin and IVD Isolation

Hairless skin and IVD tissue samples were isolated from DOX-treated and control animals, as reported before [13]. IVDs were separated from the bilateral vertebral bodies [51,72] before NP and AF segregation, as previously described [73]. Tissue samples (100 mg each) were chopped and digested with 3 mg/mL collagenase in a total volume of 2 mL for 16 h under continuous shaking. Digested tissue lysates were centrifuged at 14,500× *g* for 30 min and supernatants were processed for SPE treatment and LC-HRMS/MS analysis, as described below.

### 4.6. Preparation of Samples for LC-HRMS/MS Analysis

Calibration solutions and extracted samples of blood plasma, skin, AF and NP were spiked with 1 mL HCl (0.1 M). They were subsequently vortexed, left for 10 min and centrifuged (10,000 rpm, 10 min). The supernatant was used for SPE treatment. Initially, the SPE cartridge was conditioned with MeOH (3 mL) and water (3 mL). Afterwards, the solutions and samples were loaded on the SPE cartridge, and subsequently washed with water (2 mL). SPE cartridges were then dried for 15 min (air under vacuum). The analyte was eluted with 3 mL MeOH. The extract was dried under a gentle stream of nitrogen at 40 °C. The residue was reconstituted with 200 μL ACN:H_2_O (50:50) and sonicated in a water bath for 5 min (Bandelin Sonorex Super RK 106).

A matrix-matched calibration curve was prepared for each type of matrix (blood plasma, skin tissue, NP and AF tissue) by spiking a predefined amount of matrix with increasing amounts of DOX.

For each type of matrix, three samples for quality check were prepared at different DOX concentrations near the expected LOQ at the middle of the calibration curve dynamic range, and at high concentration.

### 4.7. Instrumentation for the Determination of DOX

#### LC-HRMS/MS Analysis

A Dionex UHPLC system (Thermo Scientific, Bremen, Germany) coupled to a QExactive benchtop Orbitrap-based high-resolution mass spectrometer HRMS/MS (Thermo Scientific) was used for the chromatographic separation, identification and quantification of the target analyte. Details about the chromatographic system, the analytical method and validation characteristics are given in the Supplementary Material.

### 4.8. Establishment of Primary Rabbit IVD Cells and Cell Culture Conditions

Primary rabbit IVD cell strains have been established in the past and were retrieved for the current study from a pre-existing cell bank maintained in the Laboratory of Cell Proliferation and Ageing, NCSR “Demokritos” [13]. In brief, AF was isolated from the NP based on the tissues’ distinct morphological and structural features, while the transition zone was discarded to avoid isolation of mixed populations of cells. Tissues were sliced and subjected to collagenase digestion (AF: 3 mg/mL, NP: 1 mg/mL), as has been described earlier [73] and the released cells were recovered by centrifugation. AF and NP cells were routinely cultured in Dulbecco’s modified Eagle’s medium (DMEM) supplemented with penicillin (100 U/mL), streptomycin (100 mg/mL) (all from PAN Biotech, Aidenbach, Germany) and 10% (*v*/*v*) fetal bovine serum (FBS, Gibco BRL, Invitrogen, Paisley, UK) in a humidified atmosphere of 5% CO_2_ at 37 °C. Cells were subcultured when confluent using a trypsin/citrate (0.25%/0.30% *w*/*v*) solution.

### 4.9. Estimation of Cell Viability

Putative cytotoxic effects of DOX on primary rabbit AF and NP IVD cells were assessed by the MTT assay, as reported previously [74]. In detail, confluent cultures were exposed to DOX concentrations from 0 to 10 μM. Following a 72-h incubation, the culture medium was aspirated and replaced by a 1 mg/mL MTT solution in serum-free, phenol-red-free DMEM (PAN Biotech) for a further 4-h incubation. Formed MTT formazan crystals were solubilized in isopropanol, and optical density at 550 nm (reference wavelength 650 nm) was measured using a FLUOstar Optima (BMG Labtech, Ortenberg, Germany) microplate reader. OD_550_ values of treated samples were divided by the respective values of the untreated samples, and data are presented as a % ratio of the untreated control.

### 4.10. Measurement of Intracellular ROS Levels

Intracellular ROS levels were estimated using the DCFH-DA assay [75]. Primary rabbit AF and NP IVD cells were plated in a 96-well plate in DMEM supplemented with 10% (*v*/*v*) FBS until confluence. Cells were pre-incubated with 10 μM of DCFH-DA for 1 h before DOX addition at final concentrations ranging from 0 to 10 μM and were further incubated at 37 °C. Cells treated with 400 μM of H_2_O_2_ served as the positive control [76]. Measurements (excitation wavelength: 485 nm, emission wavelength: 520 nm) were taken at several time-points up to 72 h using a FLUOstar Optima microtiter-plate photometer. ROS production was expressed as a % ratio of the untreated control.

### 4.11. Western Blot Analysis

Protein isolation was performed as reported previously using a Laemmli sample buffer supplemented with protease and phosphatase inhibitors (Sigma) [75]. Antibodies used here were against the following proteins: phospho-p38 (Thr180/Tyr182), p38, phospho-SAPK/JNK (Thr183/Tyr185), SAPK/JNK, phospho-p53 (Ser15) (all purchased from Cell Signaling Technology, Hertfordshire, UK), phospho-ERK1/2 (Thr202/Tyr204), panERK and p21^WAF1^ (all supplied by BD Transduction Laboratories, Bedford, MA, USA). Antibody raised against α-tubulin (used to confirm equal loading) and secondary horseradish peroxidase-conjugated antibodies (goat anti-mouse and goat anti-rabbit) were obtained from Sigma. Immune complexes were visualized using an ECL reagent (Merck Millipore, Darmstadt, Germany).

### 4.12. RNA Extraction and Real-Time PCR Analysis

For the assessment of the long-term effects of DOX on AF and NP cells in vitro, a protocol of DOX administration simulating the two-dose DOX treatment scheme applied in animals was designed (Figure 2C). Two DOX concentrations were used: one corresponding to the residual in the animals’ blood plasma DOX concentration (30 nM) and one supraphysiological concentration serving as a positive control (500 nM). Total RNA was extracted using Trizol (Invitrogen) according to the manufacturer’s instructions, as described previously [75]. Concentration and purity of the samples were estimated by a Nanodrop ND-1000 spectrophotometer (Nanodrop Technologies, Wilmington, DE, USA). First-strand cDNA synthesis was performed from 500 ng template RNA in a 10 μL reaction using the PrimeScript RT Reagent Kit (Takara, Tokyo, Japan) following the manufacturer’s instructions. Real-time PCR experiments (20 μL reactions) were performed as reported before using the KAPA SYBR FAST qPCR kit (KAPA Biosystems, Boston, MA, USA) in an Mx3000P qPCR Systems Cycler accompanied by the MxPro QPCR Software v4.10 (Stratagene, La Jolla, CA, USA) [75]. Glyceraldehyde-3-phosphate dehydrogenase (GAPDH) served as the reference gene, and relative differences in mRNA levels were estimated using the 2^−ΔΔCt^ method [77]. Primers used in the current study are presented in Table 2.

### 4.13. Immunofluorescence Experiments for the Estimation of Nuclear BrdU Incorporation

Given that one of the main characteristics of senescent cells is the loss of proliferative capacity, the long-term effect of DOX on the proliferative potential of rabbit IVD cells in vitro (following the experimental setup illustrated in Figure 2C) was assessed by immunofluorescence analysis for the estimation of nuclear BrdU incorporation, as previously described [78]. In detail, rabbit AF IVD cells exposed to DOX twice with a 7-day interval were allowed to attach onto glass coverslips after one and two subcultures 7 days post-second DOX addition before labeling with 50 μM BrdU for 48 h. Cells were then fixed with 4% (*v*/*v*) formaldehyde in phosphate buffered saline (PBS) for 10 min, permeabilized with 0.2% (*v*/*v*) Triton X-100 in PBS for 10 min and incubated with 2 N HCl for 30 min. After a 30-min blocking step with 0.5% (*v*/*v*) gelatin, samples were incubated overnight with an anti-BrdU-FITC antibody (BioLegend, San Diego, CA, USA) at 4 °C. Counterstaining was performed with 2 μg/mL 4′,6-diamidino-2-phenylindole (DAPI) dihydrochloride in PBS for 15 min at room temperature, and samples were observed under a Zeiss Axioplan 2 fluorescent microscope (Zeiss, Jena, Germany).

### 4.14. Staining with Crystal Violet

The long-term effect of DOX on the proliferation of rabbit IVD cells (following the experimental setup shown in Figure 2C) was also assessed using the colony formation assay, performed as previously reported [78]. In brief, rabbit AF IVD cells were treated with 30 and 500 nM DOX once per week for two weeks. Seven days post-second DOX administration, cells were subcultured twice, plated onto the surface of 6-well plates at a very low density and incubated at 37 °C and 5% CO_2_ for 14 days. Single cells and formed colonies were observed under a Nikon ECLIPSE Ts2 inverted microscope (Nikon, Tokyo, Japan) after fixation with 4% (*v*/*v*) formaldehyde in PBS for 30 min, staining with 0.01% (*w*/*v*) crystal violet in double-distilled (dd) H_2_O for 1 h and a wash with ddH_2_O to remove the excess of the dye. Photographs of single cells/colonies were taken using a Basler microscopy camera and software for image acquisition (Basler, Ahrensburg, Germany).

### 4.15. Sirious Red and Alcian Blue Staining

Sirius red staining was performed to determine the amount of collagen production in untreated and DOX-treated rabbit AF IVD cells and was conducted as previously reported [79]. Alcian Blue staining was performed to assess glycosaminoglycans’ content in untreated and DOX-treated rabbit NP IVD cells following a protocol described before [80]. In detail, cells were seeded in 12-well plates and grown until they reached confluence. Confluent cultures were treated according to the experimental setup shown in Figure 2C before fixation and staining. Cultures subjected to the same treatment in the absence of DOX served as the reference control cultures.

*Sirius red staining:* Cells were washed with PBS, fixed with 70% (*v*/*v*) ethanol for 30 min and washed again thrice. Deposited collagen was stained with 0.1% Sirius Red (0.1 g Sirius Red F 3B in 100 mL 1.3% picric acid solution) for 1 h at room temperature, and excessive stain was washed. Solubilization of collagen-bound stain was performed with 400 μL of 1:1 (vol/vol) NaOH 0.1% and absolute MeOH for 30 min at room temperature. One hundred microliters of solubilized stain from every sample in triplicate were transferred into the wells of a 96-well plate, and absorbance was measured at 540 nm using a FLUOstar Optima microtiter-plate spectrophotometer.

*Alcian Blue staining:* Cells were fixed with 4% (*v*/*v*) formaldehyde solution (Sigma) for 30 min at room temperature, rinsed twice with PBS and stained with 1% (*w*/*v*) Alcian Blue stain in 0.1 N HCl for 30 min. Cells were then rinsed three times with 0.1 N HCl and once with distilled water to neutralize acidity. Alcian Blue stain was extracted with 350 μL of 6 M guanidine hydrochloride (Sigma) for 20 min at room temperature. One hundred microliters of extracted stain from every sample in triplicate were transferred into the wells of a 96-well plate, and the extract’s absorbance at 620 nm was measured using a Spark Tecan microplate photometer (Tecan Trading AG, Männedorf, Switzerland).

Collagen amount and glycosaminoglycans’ content normalized to cell number were expressed as a % ratio of the control.

### 4.16. Histological Analysis

Formalin-fixed and paraffin-embedded rabbit IVDs derived from the lumbar spine of animals treated as shown in Figure 2B were used. Discs were carefully separated from adjusted endplates and further fixed in 10% phosphate-buffered formalin saline for 24 h. Fixed discs were dehydrated in degraded alcohols and xylene and then embedded in paraffin blocks. Five μm histological sections were obtained and mounted on microscopic slides. Samples were stained with hematoxylin or eosin for histology analysis. Sections were examined under a transmitted light microscope. Digital photos were obtained using a ×20 objective lens under an ECLIPSE Ts2 inverted microscope using a Basler microscopy camera and software for image acquisition.

### 4.17. Statistical Analysis for Biological Methods

Data presented regarding biological experiments stem from at least three independent experiments, and numerical values are the means ± standard deviations. Differences were considered statistically significant when *p* < 0.05 (Student’s *t*-test or ANOVA followed by Tukey’s test).

## Figures and Tables

**Table 1:** Analytical performance characteristics of the method developed for the determination of DOX in rabbit blood plasma, skin, AF and NP tissues.

	Blood Plasma	Skin	AF	NP
linear range	5–200 nM	5–800 ng/g	5–200 ng/g	5–200 ng/g
linearity R^2^	0.977	0.975	0.944	0.982
% mean rec/% rsd	78.9/6.6	79.9/5.5	69.8/4.6	71.6/10.8
single day % rsd	3.0–8.6	6.5–7.9	5.5–6.8	7.9–9.9
two day % rsd	7.6–11.9	11.2–16.7	9.9–11.4	17.2–19.0
Q3/Q1 * (% rsd) at Qc level	0.890 (22.6)	0.780 (11.7)	0.750 (17.9)	0.755 (11.4)
Q2/Q1 * (% rsd) at Qc level	0.612 (18.3)	0.590 (9.1)	0.550 (20.2)	0.558 (19.1)
LOD	0.2 nM	3 ng/g	4 ng/g	4 ng/g
LOQ	0.6 nM	9 ng/g	12 ng/g	12 ng/g

* Ion ratios (expressed as mean areas of chromatogram peaks) at the concentration of quality control (Qc), with their respective relative standard deviations. LOD: limit of detection, LOQ: limit of quantification.

**Table 2:** Primers’ sequences (5′→3′).

Target Gene	Forward Primer	Reverse Primer
MMP1	ATA-AAT-AAT-GGC-TAA-GGA-AGG-C	CAG-GAT-GAT-GTG-AGT-GAC-T
MMP3	AAT-TGT-TCA-ACA-CTT-AGG-ACT-T	TCC-AGT-TAG-ATA-CAC-AGT-TCA
MMP13	GTT-GGA-CCT-GTA-GGC-TAT-T	TCT-GGT-AGA-TGG-TTT-CCT-TTA
TIMP1	AGC-AGA-GCC-TGC-ACC-TGT-GT	CCA-CAA-ACT-TGG-CCC-TGA-TG
TGFB1	AAG-GGC-TAC-CAC-GCC-AAC-TT	CCG-GGT-TGT-GCT-GGT-TGT-AC
COL1A	ATG-GAT-GAG-GAA-ACT-GGC-AAC-T	GCC-ATC-GAC-AAG-AAC-AGT-GTA-AGT
COL2A1	AAG-AAC-TGG-TGG-AGC-AGC-AAG-AG	ATG-GAA-GCC-GCC-GTT-GAT-GG
ACAN	GGT-CGT-GGT-GAA-AGG-TGT-TGT-G	CTG-GTG-GAA-GCC-ATC-CTC-GTA-G
p53	CCA-GCC-TCT-TAG-TGA-CAA	AAA-TTG-ACC-CTG-AGC-ATT-G
p16^INK4a^	CAG-ACA-CTC-CGA-ACT-CAA	CTA-AGA-AAG-CAG-GGA-AGA-AC
GAPDH	TCC-TGG-TAT-GAC-AAC-GAA-T	GGT-TTG-AGG-GCT-CTT-ACT

## Data Availability

All data supporting reported results can be found within the article and the Supplementary Material.

## Supplementary Materials

The following supporting information can be downloaded at: https://www.mdpi.com/article/10.3390/ijms26157386/s1.

## Abbreviations

The following abbreviations are used in this manuscript:

ACN	acetonitrile
AF	annulus fibrosus
AGC	automatic gain control
ANOVA	analysis of variance
BrdU	bromodeoxyuridine
CEP	cartilaginous endplate
DAPI	4′,6-diamidino-2-phenylindole
DCFH-DA	2′,7′-dichlorodihydrofluorescein diacetate
dd	double-distilled
DMM	destabilization of the medial meniscus
DOX	doxorubicin
ECM	extracellular matrix
ERKs	extracellular signal-regulated kinases
FA	formic acid
FBS	fetal bovine serum
GAPDH	glyceraldehyde-3-phosphate dehydrogenase
IVD	intervertebral disc
JNKs	c-Jun N-terminal kinases
LC-HRMS	liquid chromatography-high resolution mass spectrometry
LD_50_	median lethal dose
LOD	limit of detection
LOQ	limit of quantification
MAPK	MAP kinase
MMP	matrix metalloproteinase
MRM	multiple reaction monitoring
MTT	methylthiazolyldiphenyl-tetrazolium bromide
NP	nucleus pulposus
OA	osteoarthritis
PBS	phosphate buffered saline
QC	quality control
ROS	reactive oxygen species
SIPS	stress-induced premature senescence
SPE	solid phase extraction
TGF-β1	transforming growth factor β1
TIMP1	tissue inhibitor of metalloproteinase 1
