# Supplementary material for: Accumulation Kinetics and Biological Action of Doxorubicin in Rabbit Intervertebral Discs
Source: Int J Mol Sci. 2025 Jul 30;26(15):7386. doi: 10.3390/ijms26157386 (PMC12347939; doi:10.3390/ijms26157386)

SUPPLEMENTARY MATERIAL

Instrumentation and methods used for the separation, identification and quantification of DOX in various matrices

LC-HRMS/MS analysis

A Dionex UHPLC system (Thermo Scientific, Bremen, Germany) coupled to a QExactive benchtop Orbitrap-based high resolution mass spectrometer HRMS/MS (Thermo Scientific), was used for the chromatographic separation, identification and quantification of the target analyte. The system consisted of a vacuum degasser, a high-pressure binary pump, an autosampler with a temperature-controlled sample tray set at 7°C and a column oven set at 30°C. Chromatographic separation was performed at 30°C using a Zorbax Eclipse Plus C18 column (100 × 2.1 mm i.d., 1.8 μm particle size; Agilent Technologies). The mobile phase consisted of 5 mM ammonium formate in 0.02% formic acid (solvent A) and a mixture of acetonitrile:water (90:10 v/v) containing 5 mM ammonium formate and 0.02% formic acid (solvent B). A gradient elution program was employed at a constant flow rate of 0.2 mL/min with solvent B starting at 5% for 3 min, initially increasing to 30% in 4 min, then increasing to 90% in 11 min and finally, set back to 5% in 11.5 min. Post-run equilibrium time was 3.5 min. The injection volume was 20 μl. Diverter valve was programed to send LC eluent in waste for the first 4 min.

A QExactive benchtop Orbitrap-based mass spectrometer (Thermo Scientific) operated in the positive polarity mode equipped with a heated ESI (HESI) source. Source parameters were: sheath gas (nitrogen) flow rate, auxiliary gas (nitrogen) flow rate and sweep gas flow rate: 40, 10 and 1 AU, respectively, capillary temperature: 250°C, ESI heater temperature: 20°C, spray voltage: +4.0 kV (positive polarity). The instrument operated in full scan mode from m/z 100-1000 at 17,500 resolving power and injection time of 100 ms and in MS/MS mode from m/z 100-1000 at 17,500 resolving power and injection time of 62 ms (product ion mode). The automatic gain control (AGC) was set at 10E6 ions. Mass calibration of the Orbitrap instrument was evaluated in both positive and negative modes weekly and external calibration was performed prior to use following the manufacturer's calibration protocol.

DOX identification was achieved by: (a) tR of compounds, (b) accurate mass of precursor *m/z* 544.1813 and (c) two characteristic precursor/product ion transitions (Q1 *m/z* 379.0817, Q2 *m/z* 397.0918 and Q3 m/z 130.0863). Q1 was the transition used for quantitation. Other diagnostics fragment ions were: *m/z* 361.0705 and 321.0747.

LC-HRMS/MS - Analytical method validation

Linearity and dynamic range of measurement, specificity, trueness, precision and limits of detection/quantification (LOD/LOQ) were determined. Blank samples were analyzed to ensure method

specificity. Trueness and precision were evaluated by analysis of water samples spiked with the analytes at the concentration level of 20 nM. For method repeatability and reproducibility, samples were analyzed in five replicates each day for two different days (n = 10). LODs were estimated from repeated measurements (n = 8) of standard solutions (10 μg/L) using the formula: LOD = t(n−1,0.95)× SD, where t(n−1,0.95) was the t-test value for n–1 degrees of freedom at 95% confidence level, (1.895 for n = 8) and SD was the standard deviation of measurements. Limits of quantification (LOQ) were estimated as 3 x LOD. In all cases, S/N values of the obtained peaks were higher than 3.

Supplementary Figure S1. Overlayed chromatograms of DOX calibration curve in rabbit blood plasma and calibration curve obtained.

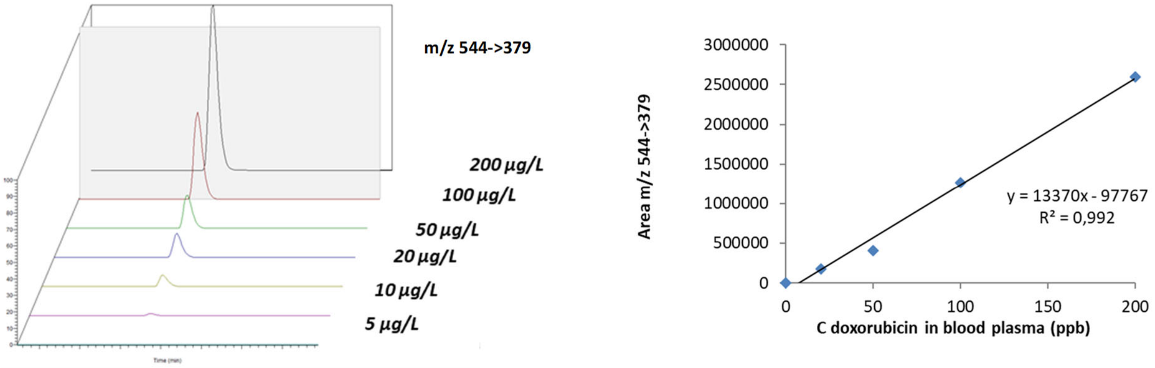

Supplementary Figure S2. Stability of DOX (50 nM) under collagenase treatment.

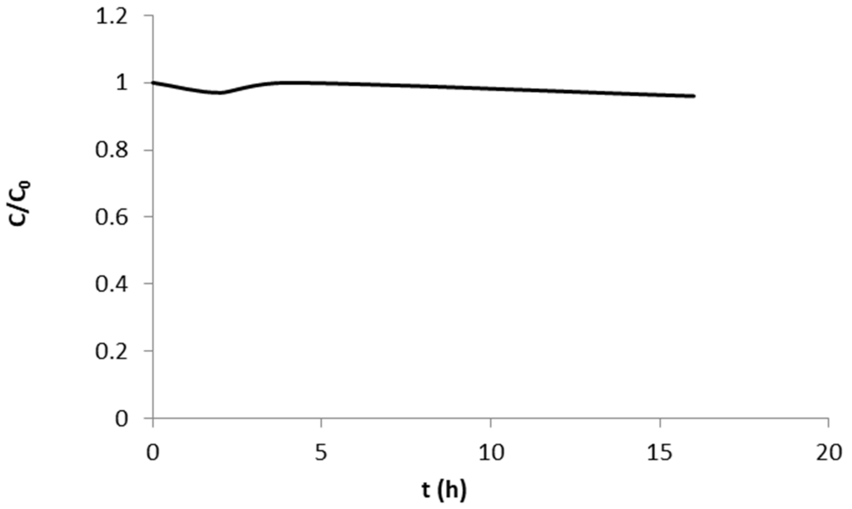

Supplementary Figure S3. MRM chromatograms of DOX in rabbit blood plasma 1h post-administration.

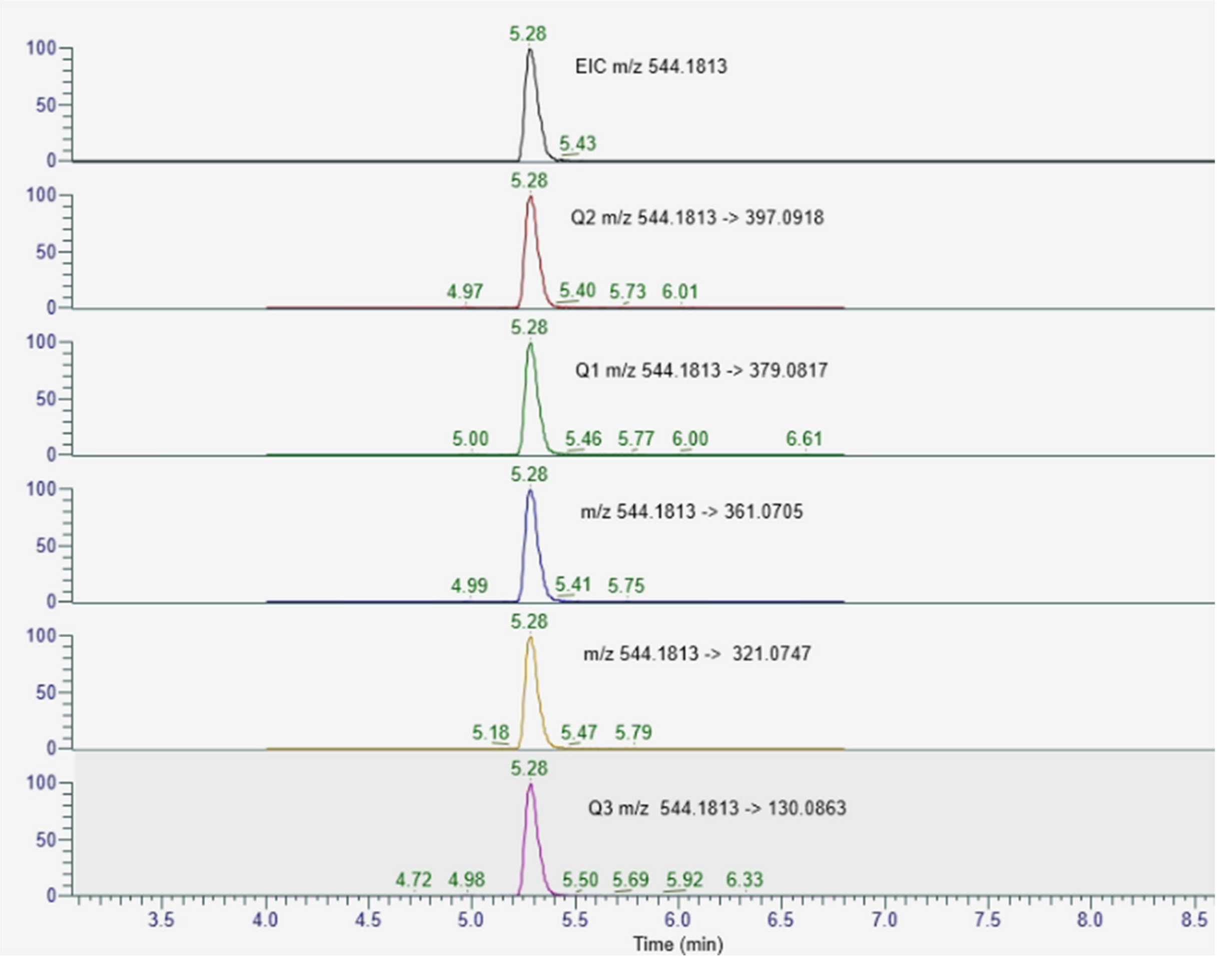

Supplementary Figure S4. MRM chromatograms of DOX in rabbit skin 1 h post-administration.

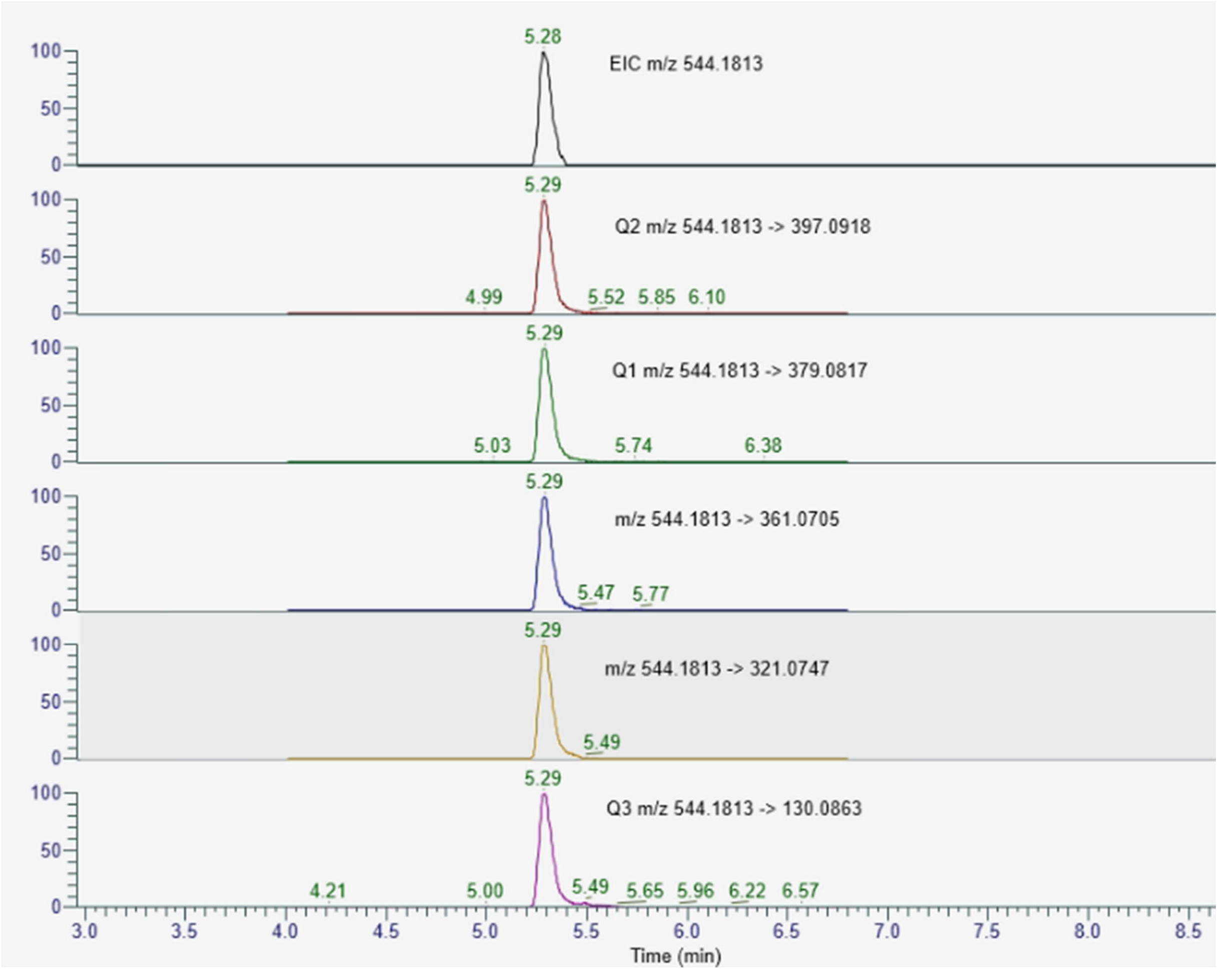

Supplementary Figure S5. MRM chromatograms of DOX in rabbit AF tissue 24 h post-administration.

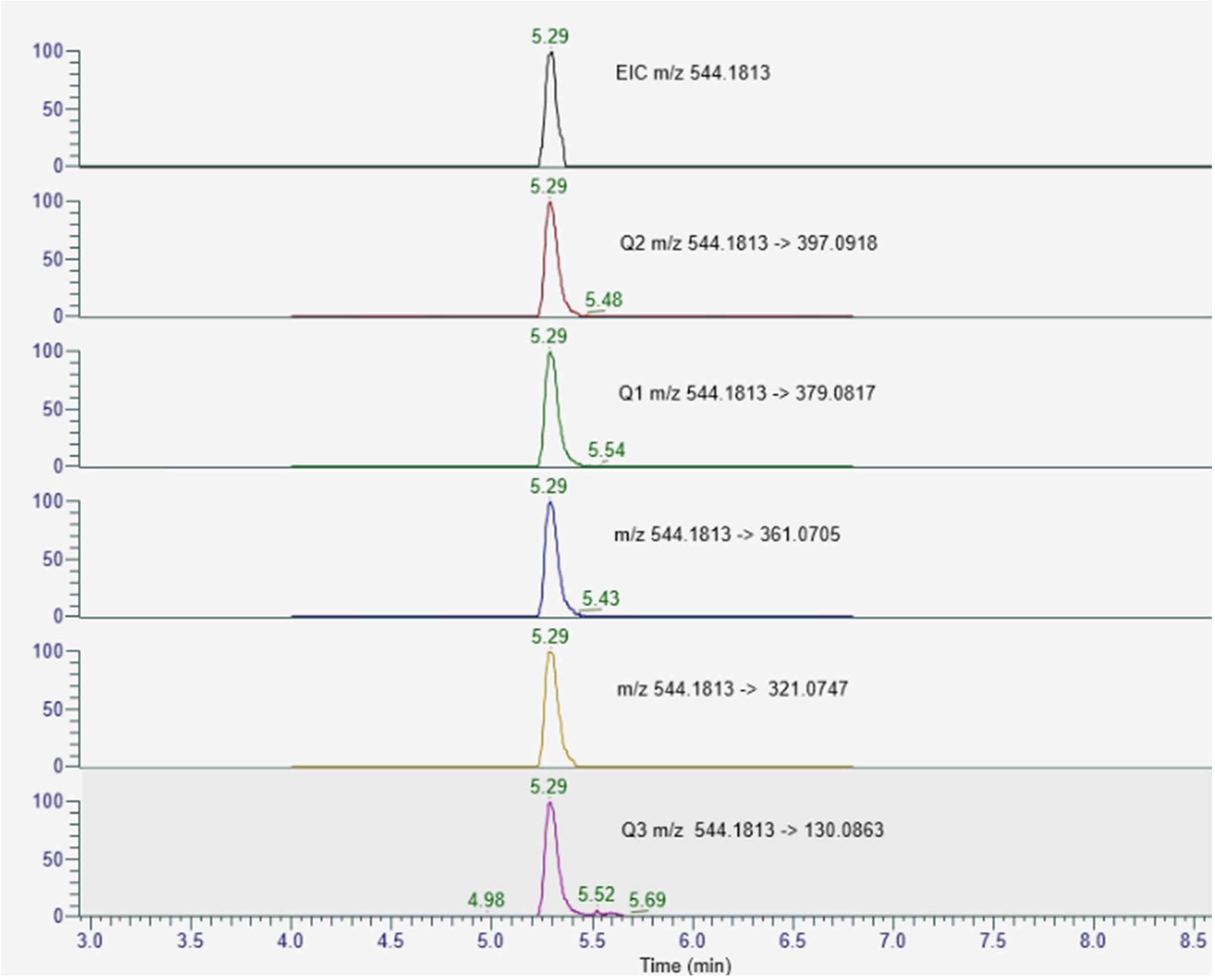

Supplementary Figure S6. MRM chromatograms of DOX in rabbit NP tissue 24 h post-administration.

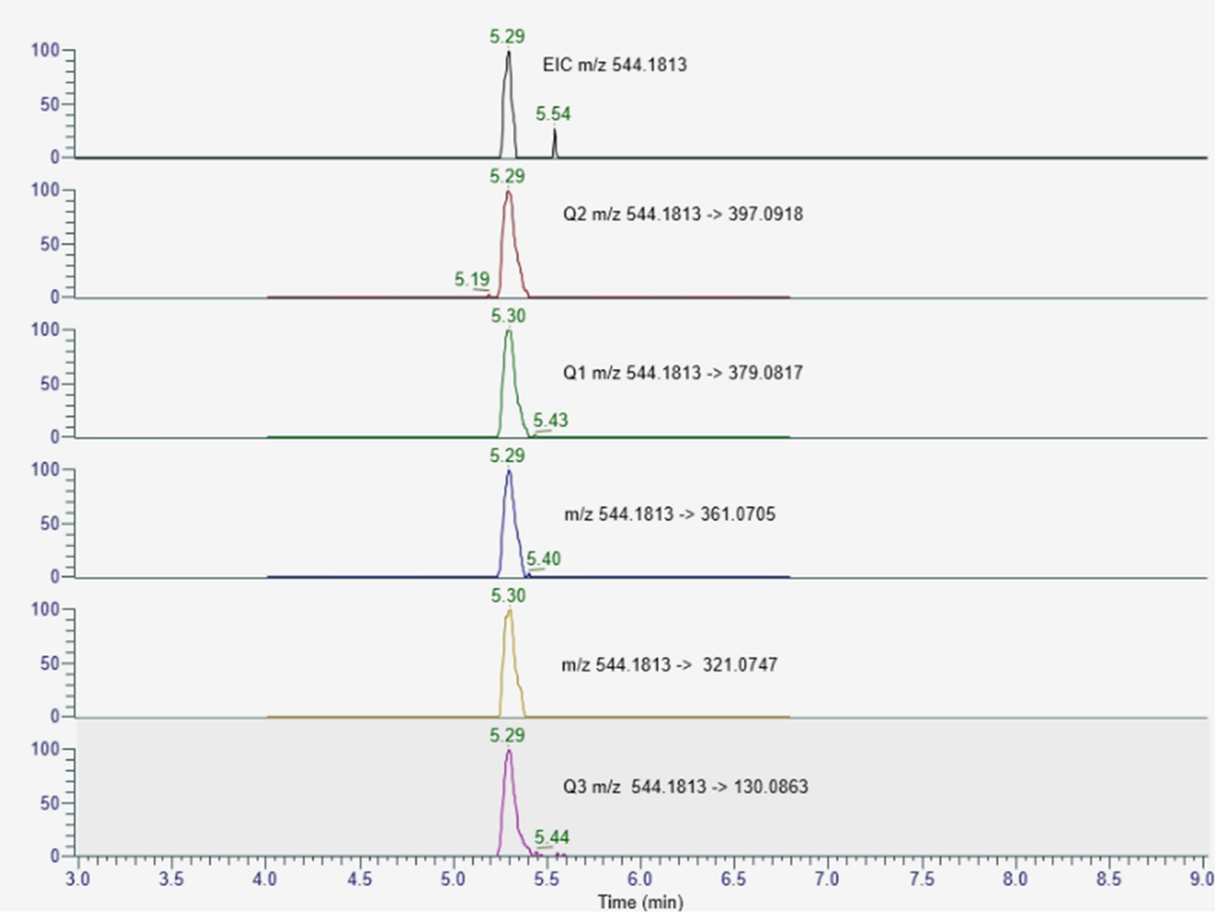

Supplementary Figure S7. Western blot analysis of DOX-treated rabbit AF IVD cells' extracts.

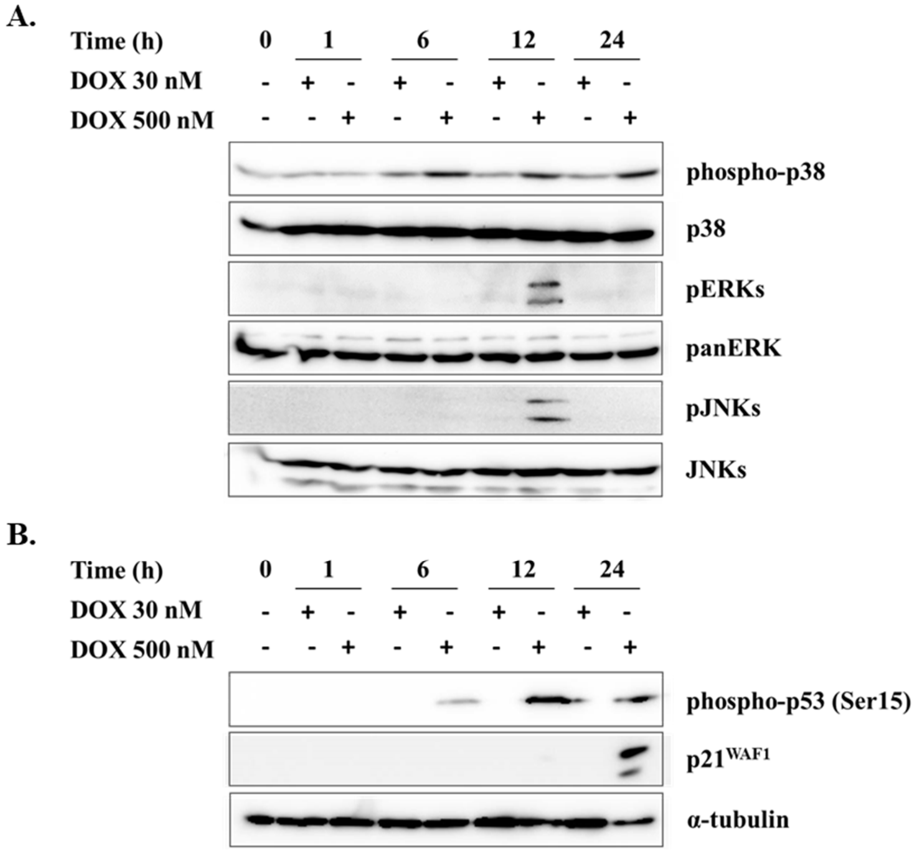

Supplement: Supplementary file 1 [file ijms-26-07386-s001.zip › ijms-3744108-supplementary.pdf]
